# Potential Crosstalk between the PACAP/VIP Neuropeptide System and Endoplasmic Reticulum Stress—Relevance to Multiple Sclerosis Pathophysiology

**DOI:** 10.3390/cells12222633

**Published:** 2023-11-15

**Authors:** Minduli Withana, Alessandro Castorina

**Affiliations:** Laboratory of Cellular and Molecular Neuroscience (LCMN), School of Life Sciences, Faculty of Science, University of Technology Sydney, Sydney, NSW 2007, Australia; umayaminduli.withana@student.uts.edu.au

**Keywords:** pituitary adenylate cyclase-activating polypeptide (PACAP), vasoactive intestinal peptide (VIP), endoplasmic reticulum (ER) stress, unfolded protein response (UPR), multiple sclerosis, neuroinflammation, microglia

## Abstract

Multiple sclerosis (MS) is an immune-mediated disorder characterized by focal demyelination and chronic inflammation of the central nervous system (CNS). Although the exact etiology is unclear, mounting evidence indicates that endoplasmic reticulum (ER) stress represents a key event in disease pathogenesis. Pituitary adenylate cyclase-activating peptide (PACAP) and vasoactive intestinal peptide (VIP) are two structurally related neuropeptides that are abundant in the CNS and are known to exert neuroprotective and immune modulatory roles. Activation of this endogenous neuropeptide system may interfere with ER stress processes to promote glial cell survival and myelin self-repair. However, the potential crosstalk between the PACAP/VIP system and ER stress remains elusive. In this review, we aim to discuss how these peptides ameliorate ER stress in the CNS, with a focus on MS pathology. Our goal is to emphasize the importance of this potential interaction to aid in the identification of novel therapeutic targets for the treatment of MS and other demyelinating disorders.

## 1. Introduction

Multiple sclerosis (MS) is an immune-mediated disorder primarily affecting the young adult population. In 2020 alone, the incidence of this disease was at an astounding 2.8 million people worldwide, with a sharp rise in number recorded from 2013 and an estimated 30% rise globally [1]. Pathologically, people with MS present multiple foci of demyelination within the central nervous system (CNS) and ongoing signs of chronic inflammation, which results in the formation of plaques/lesions [2]. Clinically, this is manifested as a plethora of varying symptoms including debilitating somatic (motor and sensory) and cognitive dysfunctions and neurological deficiencies; most common disease courses identified are relapsing–remitting MS (RRMS, approximately 87%), in which the initial attack is followed by relative periods of remission that are followed by flare-ups (relapses); RRMS is accompanied by the destruction of myelin and axonal fibers by immune cells and the presence of CNS lesions [2]. Almost 65% of people with an initial diagnosis of RRMS will progress to secondary progressive MS (SPMS), a more severe form of disease where remission periods are progressively shorter until they completely disappear [2,3]. Lastly, another clinical MS entity is primary progressive MS (PPMS). This MS subtype accounts for approxmately 10–15% of cases, and afflicted patients exhibit a steady and gradual worsening of neurological function from the onset of the disease. Interestingly enough, PPMS is generally marked by lesser CNS lesions than RRMS, although the prognosis is often more severe [2,4].

MS is a complex, multifactorial disease. Underlying contributing factors are considered to be genetic, potentially diet related, environmental, and/or based on unhealthy lifestyles. Although the exact influence of specific dietary interventions on MS progression have not been identified thus far [5], several studies suggest that dietary changes that include antioxidants can elicit some beneficial effects [6]. Considering the accumulation of reactive oxygen species (ROS), which induces oxidative stress, has been suggested as a mediator of demyelination [7], the absence of dietary antioxidative factors may favor immune cell hyperactivity, a phenomenon that can exacerbate the demyelination process [6]. For instance, in murine experimental autoimmune encephalitis (EAE) models of MS, the use of the antioxidant curcumin dampened MS-like symptoms and infiltration of the CNS by inflammatory CD3 and CD4 lymphocytes [5,6,8,9]. However, further investigations in prospective clinical studies are required in order to determine any causative link between nutrition, lifestyle and MS etiology [6]. As such, although it is understood that MS pathogenesis is surrounded by a combination of abnormalities of the immune and myelin repair systems, the precise pathogenesis of MS is yet to be fully elucidated [10,11]. Nonetheless, ER stress has been found to be a key player in several pathological domains of MS pathogenesis [12,13] several of which are ameliorated by the activation of the naturally occurring PACAP/VIP neuropeptide system [14,15,16].

In this review article, we will discuss the role of ER stress in MS pathogenesis, define the ameliorative effects of the PACAP/VIP system on myelin cell survival and self-repair and finally describe potential pathological domains for interaction between this protective neuropeptide system and ER stress. This approach may help with the identification of novel protective mechanisms of PACAP and VIP to reduce oligodendrocyte loss and perhaps promote myelin repair in chronic demyelinating pathologies such as MS, whilst also offering new opportunities for therapeutic intervention.

## 2. Multiple Sclerosis

### 2.1. Pathophysiology of MS

The etiology of MS is not fully clear; however, the disease finds its roots in the aberrant activation of the immune system against specific myelin components, leading to the invasion of the CNS by peripheral macrophages and other immune cells [2]. Specifically, activated forms of various subsets of CD4+ T helper lymphocytes, CD8+ cytotoxic lymphocytes and peripheral macrophages are recruited through the blood–brain barrier (BBB) [17]. Inflammatory factors (cytokines and chemokines) released by both infiltrating and resident immune cells (microglia) trigger demyelination and loss of oligodendrocytes (OLs). Persistent activation of this detrimental cascade causes impaired axonal transmission that culminates in neurodegeneration [17].

#### 2.1.1. Role of Lymphocytes

Autoreactive T-cells recruited through the BBB exhibit myelin-specific antigens, thus can selectively attack myelin and OLs, the cells responsible for the building up of myelin in the CNS [10,18]. Amongst various T-cell subsets, T helper 17 (Th17) cells—when activated—cause the upregulation of pro-inflammatory cytokines interleukin-17 (IL-17) and IL-6. Another cell type of interest are T helper 1 (Th1) cells, the most abundant Th cell types identified especially in EAE models. These cells release large amounts of IFN-γ, a pro-inflammatory cytokine found at high yields in lesions of human MS autopsy brains; studies indicate that when transplanted, these cells are capable of triggering EAE in recipient mice. [19,20]. Considering heightened IFN-γ is strongly associated with progression of MS in patients, this may indicate a crucial role of Th1 cells in MS pathology [21].

Furthermore, studies conducted in MS patients demonstrated that most T cells located within the CNS are CD8+ cells. While the traditional role of CD8+ T cells is the killing of cells via the production of granzymes, elevated levels of IL-17-expressing CD8+ cells are rampant around active MS lesions [22]. Lastly, B cells also partake in the pathology of MS. Studies indicate that memory B cells from MS patients display elevated expressions of CD40 and Human Leukocyte Antigen (HLA)-DR markers, suggesting an increased propensity for antigen presentation by B cells in MS. Furthermore, the release of pro-inflammatory cytokines, which exacerbate inflammation, is quite significant in MS patients compared to healthy controls [23].

The residence of activated T-cells sets off the chemotaxis of additional immune cells, including B cells, dendritic cells, microglia and natural killer cells [10]. Further, IL-17 and IL-22 secreted by Th17 cells dampen selectivity of the BBB via the interruption of endothelial tight junctions, further increasing the permeability to peripheral immune cells and triggering a cascade of destructive inflammation that determines neuronal and glial cell damage in people with MS [18].

#### 2.1.2. Microglia

Microglia are immune cells responsible for the ongoing surveillance of the CNS microenvironment and aid in repair/healing processes during the acute stages of CNS injury. This is attained via the phenotypic shift of cells from a resting state (MØ) towards either a pro-inflammatory or anti-inflammatory phenotype, canonically categorized as M1 (producing pro-inflammatory cytokines) or M2 (producing anti-inflammatory cytokines) [24]. M1 microglia mainly produces pro-inflammatory cytokines such as tumor necrosis factor-α (TNF-α), IL-6, IL-18 and IL-1. In contrast, M2 microglia mainly produces anti-inflammatory cytokines IL-4, IL-13, IL-10 and TNF-β [25]. In MS lesions, most microglia present in heterogenous states, although mainly consisting of clusters of cells with a pro-inflammatory phenotype, taking over as the primary antigen-presenting cells (APCs) and thereby enhancing T-cell proliferation [25].

Importantly, under MS conditions, microglia play a chief supportive role in phagocytosis. The toxic myelin debris formed during demyelination, which typically accumulates around the active lesion sites and interrupts proliferation and functioning of OLs, is engulfed, and digested by microglia. This enables OLs to resume their interrupted functionality and rebuild the exhausted sheaths of myelin surrounding neuronal axons; a physiological process that attempts to compensate for the unwanted myelin degradation [26,27]. However, the repeated bouts of immune attacks compromise microglia phagocytic capacity, which is followed by hindered myelin self-repair, leading to the formation of scars/plaques [28,29,30]. As such, while most clinical efforts to treat MS have been focused on the development of disease-modifying treatments (DMTs) with immunosuppressive properties with the aim of reducing the underlying neuroinflammation, to date, the same progress has not been made for the development of alternative therapies targeting myelin regeneration and/or potentiate microglia engulfment properties [31].

### 2.2. Endoplasmic Reticulum (ER) Stress

The endoplasmic reticulum (ER) is a system of membrane-bound sacs and tubules (cisternae) that originate from the nuclear envelope and are located throughout the cellular cytoplasm [32]. Those lined by ribosomes along the membrane surface are referred to as rough ER while ER that are devoid of ribosomes (or smooth ER), function as reservoirs of calcium and synthesize lipids. While the production of proteins occurs within the ribosomes of the rough ER, three-dimensional folding and post-translational alteration of proteins occurs within its cisternae. This includes the construction of multi-polypeptide proteins, formation of disulfide bonds, and the glycosylation of proteins [32]. The ER thereby folds, alters, and finally transports proteins out to intracellular or extracellular targets via the formation of transport vesicles that bud from its membrane [33].

ER stress refers to the cellular condition that results from enhanced secretory demand or triggered by pathological factors. Such factors include the presence of erroneously assembled polypeptides, supraphysiological protein synthesis rate and/or neurotoxins. Furthermore, numerous factors that hamper glycosylation or disulfide bond formation are also responsible for triggering ER stress. Additional causes for ER stress include viral infections, exposure to ultraviolet radiations, nutrient or amino acid deficiency, oxidative stress, disruptions in calcium homeostasis and/or other factors not listed here [34].

### 2.3. The Unfolded Protein Response in MS

**Figure 1:**
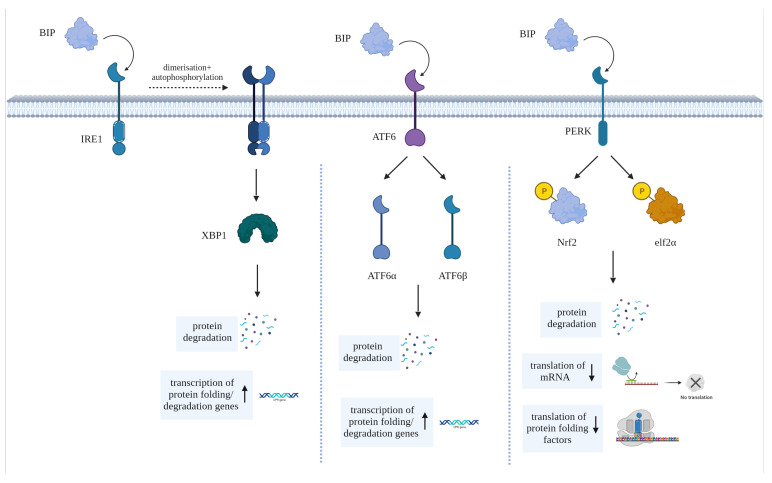
Diagram showing the main pathways activated by BIP (aka GRP78), the primary chaperone of the UPR, to elicit its effects via stress-sensors IRE1, ATF6 and PERK.

To counteract ER stress, cells activate a set of in-built signaling pathways collectively known as the unfolded protein response (UPR). As shown in Figure 1, the UPR comprises three main signaling pathways initiated by ER transmembrane proteins: Inositol-Requiring Enzyme 1 (IRE1), Protein Kinase R-like ER Kinase (PERK) and Activating Transcription Factor 6 (ATF6). These pathways lead to the expression of genes involved in chaperone protein production, ER expansion, and protein degradation [35]. This process occurs as a means of cell preservation and to promote the recovery from the erroneous production or accumulation of misfolded proteins. However, if attempts to reinstate ER homeostasis fail, UPR stimulates apoptosis [33].

The immunoglobulin heavy chain binding protein (BiP, aka GRP78)—the primary chaperone of the UPR—is endogenously bound to the ER stress-sensors PERK, ATF6 and IRE1. Upon the binding of a misfolded protein to BiP, these stress-sensors are activated, each triggering one or more pathways to contain (or reverse) ER stress [36,37]. IRE1 undergoes dimerization and auto-phosphorylation, then through its binding to X box binding protein 1, triggers the synthesis of factors that promote protein folding and upregulates ER-mediated degradation (ERAD) genes [37,38,39,40]. In contrast, ATF6 is cleaved into the two subunits ATF6α and ATF6β, which collectively trigger the synthesis of proteins that assist in the mitigation of the overload within the ER [37]. Furthermore, ATF6 also initiates the synthesis of the pro-apoptotic transcription factor C/EBP homologous protein (CHOP), which is the primary mediator of ER stress-induced cell death. Finally, the ER stress-sensor PERK phosphorylates nuclear factor erythroid 2–related factor 2 (Nrf2) and eukaryotic translation initiation factor 2 alpha subunit (elf2α) when activated; these factors suppress the translation of various proteins and upregulate the synthesis of other proteins that can either promote the correct protein folding or the degradation of erroneously folded polypeptides [41].

Importantly, in experimental autoimmune encephalomyelitis (EAE) and other murine models of MS, apoptosis of OLs has been recognized as the first hallmark of early-stage demyelinating lesions [42]. High levels of compounds that trigger inflammation, including pro-inflammatory cytokines, reactive oxygen species (ROS) and reactive nitrogen species (RNS) induce ER stress in both resident immune cells and invading hyperactivated immune cells [43]. Prolonged ER stress, in turn, causes UPR activation to counteract this process, and findings indicate that this phenomenon is highly prevalent in demyelinating lesion sites of MS; this is evidenced by elevated levels of ER stress markers CHOP, BIP and XBP-1 in a number of cell populations including microglia, OLs, T-cells and astrocytes [44] Additionally, elevated levels of inflammatory cytokines, RNS and ROS for prolonged time can trigger the UPR in both resident and infiltrating immune cells, with evidence indicating that UPR activation occurs in these cells, especially within active MS lesion sites [43]. In a study by Cunnea et al., the authors revealed that UPR markers, including ATF4, BiP and CHOP appear largely within active lesions and in the perilesional area of active lesion sites of post-mortem brains from MS sufferers [12]. CHOP and BiP levels are elevated in cells that play a central role in MS pathology, including T-cells, microglia, astrocytes and OLs; this is in conjunction with increased expression of multiple UPR markers, such as phosphorylated PERK, phosphorylated-eIF2α, BiP, and CHOP [12,43,45].

Glucosidase II is an enzyme involved in the processing of N-linked glycans during protein folding in the ER and its alpha subunit (GANAB) is involved in the activation of the UPR [46,47]; its effects involve the promotion of mRNA disintegration via the IRE1-dependent pathway. Importantly, clinical studies conducted in human peripheral blood mononuclear cells (PBMCs) demonstrated a downregulation of GANAB in interferon (IFN)β-treated humans compared to untreated controls, and an even greater GANAB downregulation in IFNβ treatment-responsive patients, thereby identifying this UPR mediator as a suitable biomarker for MS [46]. This suggests that GANAB and more broadly ER stress can be of diagnostic and prognostic significance in MS, but also in assessing the response to treatment [46,48,49].

Post-mortem analyses of human CNS tissues have demonstrated the presence of activated IRE1α and UPR in patients with Alzheimer’s disease, Parkinson’s disease and amyotrophic lateral sclerosis (ALS) [47]. However, serum levels of CHOP appeared to be similar in human RRMS patients compared to healthy controls [48]. Nonetheless, considering the limitations of data gathered from post-mortem human brains, larger studies may be needed for an appropriate interpretation of these results [48]. In addition, ER stress markers including BiP, CHOP and XBP1 along with hypoxia-related protein D-110 are also significantly elevated in post-mortem human brain lesion tissues of MS; these further evidence portray the relevance of ER stress to the clinical pathology of MS [50].

The UPR pathway poses as a potential therapeutic target in MS. Evidence suggests that tapping into this pathway could result in enhancement of OL survival and thereby increase the myelin repair potential of these cells and consequently reduce axon degeneration [33]. A study by Lin et al. discovered that in EAE models of MS, IFN-γ-specific UPR responses were protective, and that PERK activation resulted in improved OL survival [51]. Therefore, although it is well recognized that ER stress is initially neuroprotective, chronic ER stress from persistent pro-inflammatory cytokine production leads to prolonged UPR activation, leading to increased cellular apoptosis; this is confirmed by the increased levels of CHOP and is responsible for fueling disease progression [52].

### 2.4. PACAP and VIP

Pituitary adenylate cyclase-activating peptide (PACAP) and vasoactive intestinal peptide (VIP) are neuropeptides abundantly expressed throughout the CNS and peripheral nervous systems [53], where they primarily exert neuroprotective and immune modulatory roles [54]. A review by Jansen et al. (2022) detailed the mechanism of action of PACAP and VIP when binding its three receptors G protein-coupled receptors PAC1, VPAC1 and VPAC2, of which PACAP binds with significantly higher affinity to PAC1 than VIP [10].

While the effects of VPAC1 and VPAC2 activation are primarily immunomodulatory, PAC1 has been found to afford mainly neuroprotective, cell maintenance and regenerative roles by controlling the release of growth and trophic factors [55]. As such, the role of PACAP and VIP is of significant interest in the development of therapeutic strategies aimed to counteract MS pathogenesis [56]. In previous studies in vitro, using Schwann cell lines (myelinating cells of the PNS), we found that both PACAP and VIP prevent Schwann cells apoptosis [57]. In a separate study, we also identified that activation of the PACAP/PAC1 axis by exogenous administration of PACAP (or brain derived neurotrophic factor, BDNF) enhanced proteolysis by these cells, which is vital for the removal of degraded “toxic” myelin [55,58]. Schwann cells and OLs have similar functionality, it being myelin production and regeneration, with OLs found in the CNS as opposed to PNS. Considering these roles, and recent evidence suggesting that CNS infiltrating Schwann cells may also contribute to CNS myelin recovery [59], this discovery sheds light on the possible involvement of PACAP and VIP in OL survival and myelin repair, although this topic still warrants further investigations. Amongst PACAP/VIP receptors, only the PAC1 receptor is highly abundant in the CNS white matter, the primary site of injury in MS [60]. Furthermore, PAC1 expression has been identified in astrocytes, microglia, and OL progenitor cells (OPCs) [55]. Clinical studies reported no significant differences in serum PACAP between MS patients vs. healthy controls [61] Notably serum levels were downregulated more in male MS sufferers vs. females; this is interesting as male MS patients typically experience a worse prognosis than females, despite the higher prevalence of disease in the latter [62]. However, PACAP was significantly lower in the cerebrospinal fluid (CSF) of MS sufferers. Interestingly, in the same human study the downregulation of PACAP and upregulation of VIP were associated with the concurrent reduction of IL-6 in the CSF of the tested MS patients. Knowing that IL-6 is pro-inflammatory, it could be speculated that the endogenous levels of these neuropeptides may influence the course of MS [63]. These findings are in congruence with studies conducted in mouse models of MS, where PACAP treatment caused considerable improvements of clinical and pathological manifestations (lesion severity and histopathological presentation) [64]; furthermore, in vitro PACAP inhibited B7-2, a co-stimulatory molecule that exacerbates antigen presentation and suppresses Th1 cell differentiation. This, combined with findings that PACAP inhibits the synthesis of pro-inflammatory cytokines by macrophages and microglia, namely IL-1β and TNF-α in a dose-dependent manner [64], strengthen the position of the neuropeptides as potential treatment options for MS and other neuroinflammatory conditions.

In contrast to CSF findings, significantly downregulated VIP levels have been identified in the serum of patients irrespective of the MS clinical subtype (i.e., RRMS or progressive MS cases); furthermore, rising levels of VIP were positively associated with worsening disability and history of relapse [61]. However, examination of single-nucleotide polymorphisms (SNPs) of VIP in a large human study did not demonstrate any strong correlations with disease severity [65].

With respect to PACAP, despite the yet insufficient clinical evidence of therapeutic efficacy in humans, it is already established that the peptide promotes the growth of OLs and enhances OPCs proliferation, at least in murine models of MS disease. However, there is still lack of understanding of the extent and mechanisms by which PACAP and VIP modulate the myelin reparative activities of OLs [66].

### 2.5. Gap in Research

Typically, available treatments for MS are immunosuppressive, and aim to reverse the underlying autoimmunity. However, to date, there remains no cure for MS [2]. While immunosuppressive treatments are mainly symptomatic, they do not resolve the underlying consequences of repeated immune attacks, so are unable to arrest MS progression. Furthermore, little success has been obtained with myelin regenerative treatments, in most cases due to the difficulty in translating preclinical evidence to the clinic or due to the severity of side effects of tested compounds in clinical trials [67,68,69]. A drug that can effectively promote myelin repair could potentially be used in combination with currently available immunosuppressive drugs as a polytherapy to slow MS disease progression [2,67,68,69,70]. Therefore, novel therapeutic targets for MS are needed now. At present, there are no reliable drugs that target ER stress and/or the UPR machinery to improve OL survival or enhance myelin repair. Therefore, considering that the neuroprotective and immune-modulatory role of PACAP and VIP hold the potential to interfere with ER stress and its downstream neurodegenerative effects, it is not unreasonable to hypothesize that targeting this neuropeptide system could impart anti-ER stress effects in the diseased CNS. This and other aspects will be discussed in detail in the sections below.

## 3. Pathological Domains for Potential Interaction between PACAP/VIP and ER Stress

### 3.1. Direct and Indirect Effects of PACAP and VIP against ER Stress

CHOP, encoded by the gene DNA Damage-Inducible Transcript 3 (*DDIT3*) is a transcription factor involved in cellular stress responses, particularly in the context of ER stress and the UPR [71]. CHOP is one of the downstream targets of the UPR signaling pathways, specifically activated by the PERK branch [72]. This UPR effector is associated with the induction of apoptosis under severe or prolonged ER stress conditions, where it promotes apoptosis by upregulating the expression of pro-apoptotic genes and suppressing anti-apoptotic factors [73].

In a study by Mansouri et al. (2017), neuronal stem cells (NSCs) obtained from the brains of adult mice were exposed to varying concentrations of ketamine, a dissociative anesthetic. The aim of this study was to determine the effect of ketamine on NSC viability and ER stress, and to elucidate the ability of the PACAP/PAC1 axis to reverse any detrimental effects of the drug [14]. When NSCs were exposed to both ketamine and PACAP, relative ATP levels increased, and more importantly, the peptide fully prevented the reduction in cell viability, confirming the previously reported anti-apoptotic effects of PACAP [14,74,75]. Furthermore, when PACAP was replaced with the PAC1-specific agonist—Maxadilan—cells responded similarly, hence showing a reduction in the UPR effector CHOP and suggesting a PACAP/PAC1-driven attenuation of ketamine-induced ER stress [14].

The results of the study above corroborated the findings from other investigations utilizing adult NSCs exposed to hypoglycemic conditions, where PACAP administration resulted in a similar attenuation of cell death, paralleled by reduced CHOP expression levels [74]. This sparked interest because in the hippocampus of mice subjected to EAE, CHOP expression was also found to be significantly increased in OLs of immunized mice vs. controls [76]. These findings imply that treatments able to modulate CHOP expression may also be able to prevent OL cell death. Although these findings have not been investigated in clinical studies, it must be highlighted that the UPR is a highly conserved process from an evolutionary perspective. The IRE1 branch in particular remains highly preserved in almost all eukaryotic species, including yeasts, which indicates its physiological functions are likely to be conserved in all organisms, including humans [77,78]. Therefore, it can be argued that such preclinical findings could be translated into similar results in humans, although this remains to be further investigated.

An association between PACAP and ER stress has also been identified in vitro in neuronal-differentiated pheochromocytoma PC12 cell lines challenged with tunicamycin (TM), an inhibitor of protein glycosylation and potent ER stress inducer [79,80]. In this study, it was demonstrated that nanomolar concentrations of PACAP were sufficient to prevent TM-induced cell death [79]. Considering the direct ER stress-inducing effects of TM, these findings corroborate the idea that the ameliorative activities of PACAP may occur through the direct inhibition of ER stress pathways. Importantly, these neuroprotective effects were not seen in undifferentiated PC12 cells, where the relative PAC1 receptor abundance is low while that of VPAC1 and VPAC2 is similar to differentiated cells. This further evidence pinpoints the importance of the PACAP/PAC1 axis in preventing ER stress.

Combined in vitro and in vivo studies by Rat et al. (2011) in PC12 cells and using a mouse model of Alzheimer’s disease provide further evidence that PACAP administration counteracts cell loss caused by amyloid beta accumulation [81]. Treatment with the peptide induced a rapid increase in the growth factor brain-derived neurotrophic factor (BDNF) and the anti-apoptotic protein Bcl-2 [82]. BDNF has been associated with dampening of ER stress and neuroprotection against cell death in murine mouse models from prior studies, whilst Bcl-2 upregulation has multiple beneficial effects as described above. Furthermore, Bcl-2 obstructs the exit of cytochrome c into the cytoplasm, which hinders caspase-3 activation [81].

PACAP and VIP stimulate the production of inositol trisphosphate (IP3) [83], which is a second messenger involved in calcium release from the ER [84]. IP3 binds to receptors on the ER membrane, leading to the release of calcium ions into the cytoplasm [85]. PACAP/VIP-induced calcium mobilization from the ER stores into the cytoplasm, contributes not only to changes in intracellular calcium concentrations, but importantly, represents a major mechanism to mitigate ER stress via the control of ER calcium homeostasis [86]. In fact, whilst physiological levels of ER calcium facilitate protein folding, persistent perturbations or excessive depletion of ER calcium stores are key events in the abnormal induction of the UPR and ER stress [87].

These scenarios provide further indication of the neuroprotective and anti-ER stress effects of PACAP, which are likely mediated by PAC1 receptors [79]. However, current knowledge does not allow to negate the anti-apoptotic/ER stress potential of VPAC1 and VPAC2 receptors [88,89], especially that triggered by inflammatory stimuli. In fact, VIP also protects NPCs via the inhibition of pathways associated to ER stress [90]. In fact, studies show a rapid rise of the endogenous VIP transcripts and proteins in enteric neurons challenged with colchicine or in sympathetic and sensory neurons after experimental axotomy [91], further portraying the importance of the endogenous VIP signaling as an endogenous protective mechanism to regain CNS homeostatic control under neuronal ER stress triggered by microtubule disruption and/or inflammation secondary to mechanical injury. Evidence also indicates that nerve growth factor (NGF) can trigger VIP overexpression in neurons [92]. Moreover, NGF administration prevents chronic activation of the UPR and reduces neuronal cell death by restoring ER homeostasis [93], pointing to VIP as an additional therapeutic molecule able to improve cell survival of neurons, most likely by blocking ER stress pro-apoptotic pathways.

Interestingly in a separate human study, Naltrexone—an inhibitor of TM-induced ER stress that has been shown to lower CHOP and GRP78 in intestinal epithelial cell lines—reduced endoscopic inflammation in patients with irritable bowel disease (IBD), thereby displaying clinical efficacy and indicating a mechanism of action involving the dampening of ER stress to reduce inflammation [94]. These findings pertaining to the effect of ER-stress inhibitors are in congruence with human MS studies on GANAB, the enzymatic subunit involved in the UPR; as mentioned earlier, downregulation of GANAB is demonstrated in MS patients treated by IFNβ, an effect further enhanced by treatment-responsiveness [46]. Furthermore, histological analyses of synovial biopsies obtained from synovitis patients, demonstrate a strong correlation between the levels of 10 different proteins involved in ER stress (including calreticulin, a UPR chaperone and more importantly, GANAB) and the histological inflammation score obtained [95].

These findings suggest that the decline in levels of GANAB seen during successful treatment-responsive courses in MS patients are directly linked to the rise in GANAB that occurs in conjunction with elevated histological inflammation, as seen in synovial tissue [95]. In MS, we argue that this supports the elevated levels of ER stress markers found in active lesions in post-mortem human brain tissue [50]. While there is still much work to be performed to fully comprehend the potential interactions of PACAP/VIP on ER stress in humans, these findings give some hope on the therapeutic potential of this neuropeptide system, which seems to interfere with relevant anti-inflammatory mechanisms and inhibition of pathological ER stress pathways.

### 3.2. ER Stress in Immune Cells

ER stress also occurs in immune cells and plays a significant role in regulating immune functions. Resident and peripheral immune cells, such as microglia, macrophages, dendritic cells, lymphocytes, and neutrophils, are exposed to various stressors and challenges during their physiological activities, or when exposed to pathogen encounters and/or other inflammatory triggers [96]. In addition, there is evidence to suggest that ER stress in immune cells can also contribute to the production of self-reactive antibodies [97], purportedly playing a role in the pathogenesis of autoimmune disorders, including MS. In this section, we will highlight some of the main findings linking ER stress to immune cell functioning and discuss how this can be harnessed to develop therapeutic solutions able to reverse ER stress-induced immune cell alterations.

Multiple studies have demonstrated that cocaine-induced ER stress in microglia results in autophagy and elevates microglial activation [71,72,73]. In mouse models of depression, it was shown that polarized microglia exhibited concurrent signs of ER stress, which upon reversal, caused a phenotypic shift of microglia towards anti-inflammatory phenotypes [71]. Furthermore, in a study investigating the toxic effects of paraquat—a well-known herbicide used to mimic environmental Parkinson’s disease in mice—elevated ER stress signatures in microglia were associated with heightened neuroinflammation and NSC depletion, suggesting that exacerbated inflammation occurs through a mechanism involving ER stress in this glial cell population [98].

In macrophages, prolonged ER stress disrupts cellular homeostasis and promotes pro-inflammatory cytokines production [99]. Furthermore, ER stress is associated with LPS-induced production of IFNβ by these cells, in a process that correlates with the expression of XBP1 protein [100]. This suggests that inflammation and ER stress can be mutually activated in peripheral macrophages, perhaps to sustain their damaging potential once these cells have penetrated the CNS.

Various studies have also linked ER stress with the ability of dendritic cell to function as antigen-presenting cells [101,102]. In models of skin inflammation, depletion of XBP1 in dendritic cells was associated with dampening of both inflammation and release of inflammatory cytokines IL-23 and IL-6 [101]. In line with these findings, another study evidenced that IRE1α stimulation influenced IRE1α-dependent decay of MHC-1 mRNA, demonstrating that countering of ER stress results in the depletion of antigen-presentation by dendritic cells [102], although via modulating a different UPR branch.

Similarly, in in vivo and in vitro allograft rejection models, suppression of IRE1α in CD8+ T cells limited their replication, hindered cell functioning, and reduced memory activation, collectively contributing to immunosuppression [103]. Further, studies indicated that knockout of either XBP1 or PERK genes resulted in a significant reduction of granules released by neutrophils, an effect that became cumulative when both genes were simultaneously silenced [104].

As mentioned earlier, OLs are highly vulnerable to ER stress consequent to inflammation triggered by neighboring or infiltrating immune cells, especially in response to interferon (IFN)-γ [105]. In relation to MS pathology, these findings may imply that the inflammatory milieu around lesion sites may be crucial in obstructing OLs ability to (re)-myelinate CNS axons [106].

**Figure 2:**
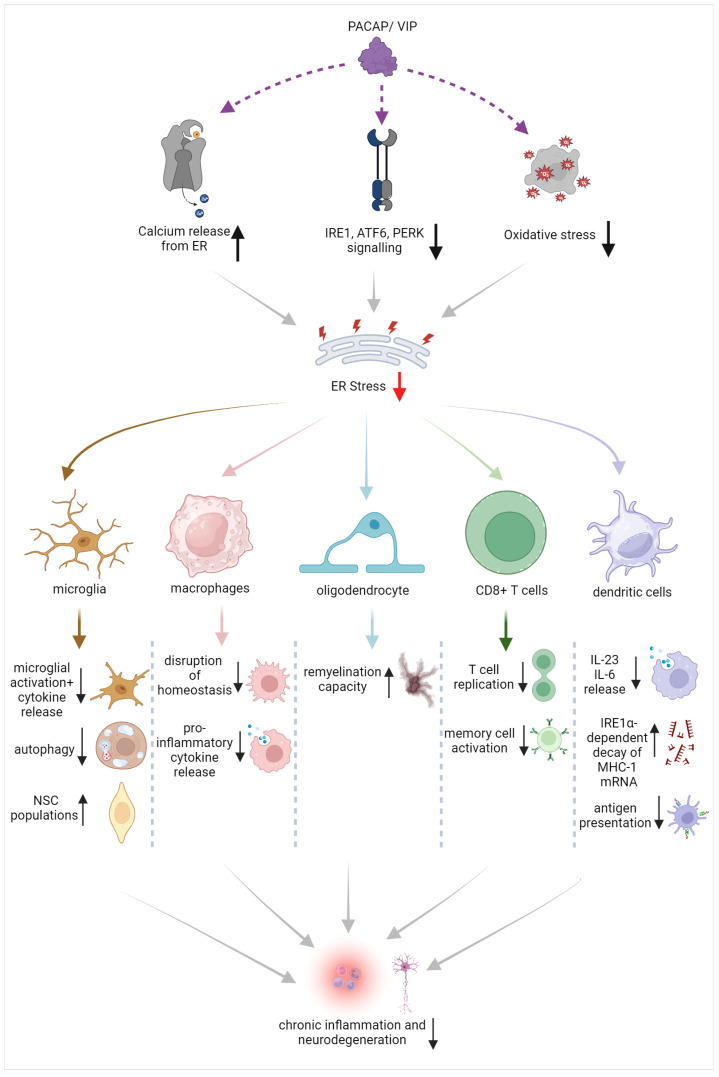
Schematic of the beneficial effects of PACAP/VIP on ER stress in different immune cell and glial populations, with suggested points of interaction and proposed mechanisms of action.

Based on the evidence provided, there is strong potential to eliminate the exacerbated and chronic activation of resident and peripheral immune cells by suppressing ER stress in immune cells [107]. This could include dampening ER stress triggers via the modulation of calcium signaling, inhibiting ER stress responses by tackling specific branches of the UPR such as XBP1 or IRE1α kinase, or by implementing antioxidative strategies [107,108]. Such activities seem to be regulated by the PACAP/VIP system (summarized in Figure 2). However, before such remedial strategies can be applied, it is paramount to first determine the therapeutic window during which UPR inhibition remains beneficial as well as defining a plan to avoid potential off-target effects in normally functioning immune cells [108,109].

### 3.3. ER Stress and the PAC1 Receptor

Findings from [110] in Neuro2a cells (a neuronal cell line) suggested that PAC1 expression is directly modulated by ER stress pathways. Under oxygen glucose deprivation (OGD), a condition known to trigger ER stress and induce ischemic damage, PAC1 mRNA and protein expression were significantly reduced [110]. TM, an ER stress inducer, further reduced PAC1 expression. In contrast, treatment with salubrinal—a specific inhibitor of eIF2α phosphatase enzymes able to activate the PERK branch of the UPR—rescued PAC1 expression levels [110], suggesting an inverse correlation between the levels of ER stress and those of the PAC1 receptor [111,112]. A possible mechanistic explanation for such inverse relationship may be inferred from a study conducted using the PC12 cell line [113]. In this study, the authors showed that in NGF-stimulated cells, Ras/MAPK pathway induction caused the translocation of the transcription factor specificity protein 1 (Sp1) to the nucleus, causing the activation of the PAC1 promotor region in neurons [113], thereby increasing its expression levels. In contrast, ER stress has shown potent inhibitory effects on Sp1 activation [114] and further evidence demonstrated that transglutaminase 2 (TG2) and Sp1 crosslinking may be at the basis of PAC1 negative regulation [110], which could explain how PAC1 expression could be downregulated as ER stress increases. However, it should be noticed that the interaction between TG2 and Sp1 can vary depending on the specific cellular environment, the presence of co-factors, and the target genes being regulated. Additionally, research on this interaction is still ongoing, so the full extent of its biological significance is still under scrutiny.

However, based on the findings above and considering that TM-induced apoptosis is prevented by PACAP, we suggest that the relationship between ER stress and PAC1 receptor regulation may be bidirectional. In other words, activation of the PAC1 receptor by PACAP may inhibit Sp1 activity (and/or TG2-Sp1 crosslinking), thereby reducing chronic activation of ER stress pathways leading to chronic inflammation (in immune cells) or cell death (in neurons). In contrast, chronic ER stress could downregulate PAC1 mRNAs, thereby reducing the endogenous protective/immune modulatory capacity of cells. In this setting, the PACAP/PAC1 axis could serve as a regulatory pathway to prevent aberrant ER stress or UPR activation, a pathway that could potentially find application as a target for the treatment of neurodegenerative disorders that exhibit overt signs of sustained ER stress, including the most severe forms of progressive MS [12].

### 3.4. PACAP/VIP Activation of cAMP-Dependent Protective Pathways and ER Stress

**Figure 3:**
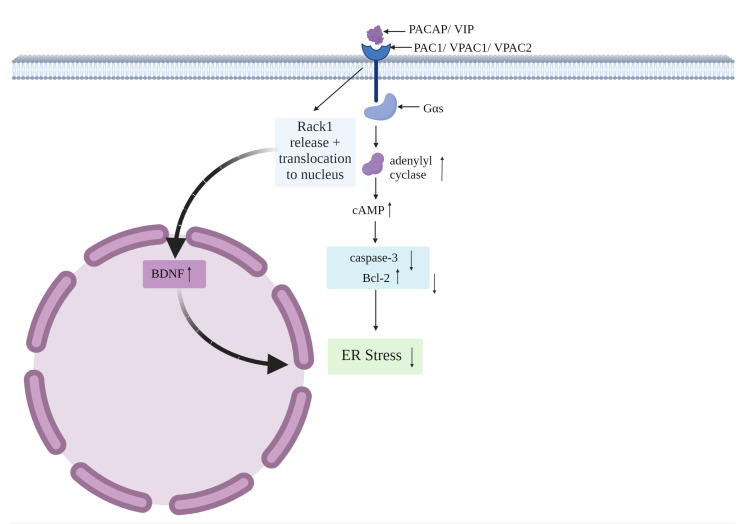
The interactions of PACAP/VIP on ER stress via the cAMP pathway.

Considering the potential points of convergence between PACAP/VIP and ER stress identified thus far, in this section we will discuss the main intracellular pathways activated by the peptides that are linked to ER stress attenuation and drive the main protective effects in the CNS. As highlighted in Figure 3 below, PACAP/VIP-induced activation of these signaling cascades target abnormal UPR exacerbations, prevent ER stress-induced apoptosis and dampen other detrimental processes (i.e., inflammation, autophagy) in the CNS. Of note, considering the commonality of receptors activated by PACAP and VIP, the similar high affinity of the peptides for VPAC receptors [115,116] and the limited in vivo studies reported on VIP, we cannot exclude that there may be a significant overlap in the intracellular pathways activated by the two neuropeptides (PACAP or VIP) to inhibit ER stress, although further work in this regard is still needed.

Firstly, PAC1, VPAC1 and VPAC2 are typically preferentially linked to Gαs, a heterotrimeric G-protein that promotes the cyclic adenosine monophosphate (cAMP)-dependent pathway via stimulation of adenylyl cyclase. The activation of adenylyl cyclase, in turn, results in increased cAMP accumulation [115]. Importantly, cAMP elevation triggers the inhibition of the apoptotic marker caspase-3, coupled with increased levels of the anti-apoptotic protein Bcl-2 [82]. Furthermore, the capacity of PACAP to release the receptor of activated C kinase-1 (Rack1) from the NMDA receptor it is bound to is well recognized [117]; this enables the translocation of Rack1 to the nucleus wherein it upregulates BDNF, who can then dampen the damaging effects of ER stress [118,119]. PACAP also suppresses expression of Bcl-2-associated X protein (Bax), a leading pro-apoptotic protein in the ER stress-induced apoptotic pathway, especially in neurons [120,121]. Considering the relevance of these molecular targets in counteracting apoptosis caused by ER stress, it is conceivable that PACAP (and perhaps VIP)-mediated activation of cAMP, reduced Bax and caspase-3 activities, along with the upregulation of Bcl-2 and BDNF, may result in the direct or indirect blockade of ER stress-induced apoptosis in the CNS [75,120,122].

### 3.5. PACAP/VIP and Microglial Oxidative Stress

Oxidative stress and ER stress are two interconnected cellular processes that have significant implications in the maintenance of microglial physiology. Indeed, oxidative stress directly modifies proteins involved in ER function [123] and disturbs calcium homeostasis [124], both of which are crucial for proper ER functioning. In contrast, ER stress itself, via the activation of the UPR, can also lead to the production of reactive oxygen species (ROS) as a byproduct [125]. As such, the interplay between oxidative stress and ER stress can create a feedback loop, where each stressor exacerbates the other, potentially leading to a vicious cycle of cellular dysfunction and damage. Understanding the influence of PACAP/VIP on microglia oxidative mechanisms could offer an additional tool to arrest this concatenation of events, hence improving microglial health.

Chronically activated microglia release significant levels of ROS, and this contributes to microglia-mediated neurodegeneration, a familiar phenomenon seen across several neurological conditions such as AD, PD, ALS and including MS [126]. Microglial cells express nicotinamide adenine dinucleotide phosphate oxidase (NOX), an enzyme complex responsible for transferring electrons from NADPH to molecular oxygen, leading to the production of superoxide anion (O_2_•^−^) and subsequently other ROS [127]. During the shift of microglia from a resting to a pro-inflammatory M1 state (but not in M2 microglia), NOX activity is remarkably increased [128], and this leads to the exacerbation of inflammation; thus, the chronic polarization of microglia or the shift from M2 to M1 phenotypes could create a loop to sustain CNS inflammation and contribute to worsen lesion severity in MS [126]. In the MS brain, activated microglia release oxidizing radicals such as nitric oxide (NO) and hydrogen peroxide, which as mentioned, contribute to further aggravate the neurodegenerative process [129]. Studies by Grey et al. (2008) indicate that in MS lesions, the lysosomal enzyme myeloperoxidase involved in the synthesis of multiple ROS is upregulated by both infiltrating macrophages and microglia [129]. Furthermore, high concentrations of oxidized lipids have been detected in myelin membranes of dying OLs in demyelinating regions of the brain, again highlighting the importance of oxidative stress in MS pathophysiology [130].

**Figure 4:**
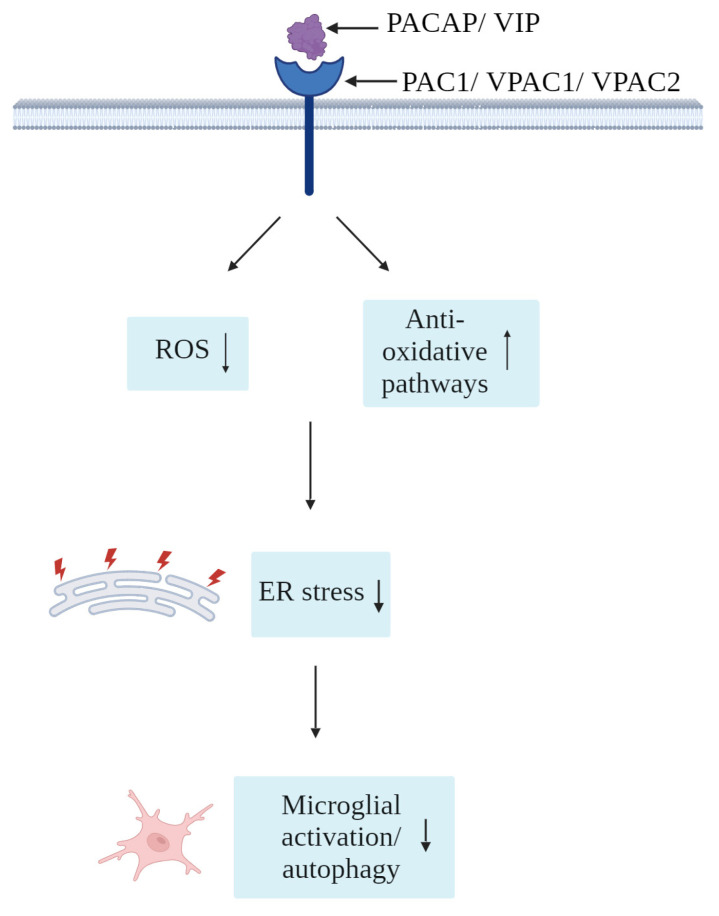
Simplified schematic of the proposed antioxidative effects of PACAP/VIP against microglial oxidative and ER stress.

As shown in Figure 4, several reports have indicated prominent roles of PACAP/VIP in the physiological regulation of oxidative stress [131], as well as potent antioxidants under experimentally induced oxidative stress [132,133]. In adult mice, administration of PACAP results in lowered levels of oxidative species and promoted the activation of antioxidative pathways [131]. In that same study, the authors show that adult PACAP null mice had impaired antioxidant potential [131]. Studies in zebrafish models of oxidative stress suggest that PACAP antioxidant activities mainly occur through the blockade of oxidative stress-induced apoptosis [134]. However, these investigations were performed in hair cells, which are specialized sensory cells that transmit electrical impulses located within minute epithelial receptor organs (neuromasts) of the inner ear of vertebrates that are particularly receptive to oxidative stress; as such, further in vivo studies may be required to validate these findings in different cell populations [134]. Similarly, as reviewed by Korkmaz and Tunçel (2018), VIP is also a potent inhibitor of oxidative stress in the CNS, with important implications on neurodegenerative diseases featuring mitochondrial dysfunctions, such as Parkinson’s disease [135]. Moreover, in rats, hypothalamic injections of VIP modulated food intake and produced metabolic changes via the reduction of NO levels [136], likely acting as an inhibitor of local NO secretion by surrounding glia. Based on these arguments, PACAP/VIP antioxidant activities in microglia (and other cell types) may also partake to the resolution of ER stress, providing a further venue of investigation for the treatment of those CNS diseases where oxidative and ER stress are the predominant pathological pathways.

## 4. Conclusions

As highlighted in this review article, the pathophysiology of MS is a complex phenomenon caused by a combination of neurodegenerative processes and chronic inflammation [137]. Exacerbated inflammation, detrimental to myelin of the CNS, is aggravated by an uncontrolled release of pro-inflammatory cytokines and other toxic mediators, a process aided by the hyperactivation of a plethora of immune cells, including Th17, Th1, CD8+ T cells, microglia, and B cells [20,22,23]. In this context, the potential influence of ER stress and aberrant UPR activation in MS pathology has been brought to light [43]. Therefore, whilst UPR holds many beneficial effects in restoring ER homeostasis, its chronicity may have several downfalls, including apoptosis of OLs and other CNS cells [138].

PACAP has demonstrated beneficial effects for MS in several studies, including an improvement of lesion severity and amelioration of disability in mouse models, and a downregulation of IL-6 in human studies, suggesting a robust anti-inflammatory mechanism of action [63,64]. Importantly, in this review, these protective effects are suggested to be indirectly linked via the inhibition of downstream pro-apoptotic effectors of different UPR branches and the elevation of anti-apoptotic molecules, or possibly directly linked to the inhibitory activities of ER stress key players. Overall, it can be implied that there remains much to explore and consider regarding the role of PACAP/VIP system in the modulation of ER stress responses produced in the demyelinating CNS [90,139]. The bidirectionality of this regulatory activity is suggested to be a plausible theory, in which chronic ER stress could downregulate the endogenous expression of PACAP/VIP receptors, thereby reducing the endogenous ability of PACAP/VIP to afford neuroprotection; this must be clarified via further experimentation.

We suggest that the influence of these neuropeptides on oxidative stress, ER calcium stores and the cAMP pathway is a potential route through which this modulation could occur [120,140]. Gaining a deeper understanding of these pathways could be relevant in enhancing OL survival and dampening activation of microglia in active lesion sites. However, further research is required to fully confirm the impact of PACAP/VIP in dampening ER stress, elucidate the mechanisms by which it could occur and, by extension, the ability to use this capacity as a therapeutic opportunity in attempts to prevent neurodegeneration in MS and perhaps other demyelinating disorders [57].

## Data Availability

No research data was generated for the preparation of this manuscript.
